# Mitochondrial transplantation combined with mitoquinone and melatonin: A survival strategy against myocardial reperfusion injury in aged rats

**DOI:** 10.1113/EP092292

**Published:** 2025-03-31

**Authors:** Behnaz Mokhtari, Mitra Delkhah, Reza Badalzadeh, Samad Ghaffari

**Affiliations:** ^1^ Alavi Aging Research Institute Tabriz University of Medical Sciences Tabriz Iran; ^2^ Molecular Medicine Research Center Tabriz University of Medical Sciences Tabriz Iran; ^3^ Department of Physiology, Faculty of Medicine Tabriz University of Medical Sciences Tabriz Iran; ^4^ Student Research Committee Tabriz University of Medical Sciences Tabriz Iran; ^5^ Cardiovascular Research Center Tabriz University of Medical Sciences Tabriz Iran

**Keywords:** ageing, combined modality therapy, melatonin, mitochondria, mitoquinone, myocardial ischaemia–reperfusion

## Abstract

Myocardial ischaemia–reperfusion (IR) injury poses a severe threat to cardiac health, particularly in the ageing population, where susceptibility to such damage is significantly heightened owing to age‐related declines in mitochondrial function, thus highlighting mitochondria as crucial targets for innovative therapies. The aim of this study was to investigate the combined modality therapy involving mitochondrial transplantation and the mitochondrial boosters mitoquinone and melatonin to address myocardial IR injury in aged rats. A total of 54 male Wistar rats, aged 22–24 months, were randomly divided into groups that either received IR injury or not, and were subjected to various treatments, both individually and in combination. Myocardial IR injury was induced by temporarily blocking and reopening the left anterior descending coronary artery. Mitoquinone was given intraperitoneally for 14 days prior to ischaemia, while melatonin and isolated mitochondria were administered intraperitoneally and intramyocardially, respectively, at the onset of reperfusion. Finally, we evaluated changes in haemodynamic indices, creatine kinase‐MB levels, mitochondrial function endpoints and the expression of mitochondrial biogenesis genes, including sirtuin 1 (*SIRT‐1*), peroxisome proliferator‐activated receptor gamma coactivator 1‐alpha (*PGC‐1α*) and nuclear respiratory factor 2 (*NRF‐2*). The triple therapy enhanced myocardial function, decreased creatine kinase‐MB levels and improved mitochondrial function along with the expression of mitochondrial biogenesis genes in aged IR rats. This combined approach elicited significant cardioprotection in comparison to single or dual therapies. The triple therapy provided substantial cardioprotection in aged rat hearts by improving mitochondrial function and biogenesis through enhanced *SIRT‐1*/*PGC‐1α*/*NRF‐2* profiles, suggesting a promising strategy for mitigating IR injury in elderly patients.

## Abstract (highlights)

- **What is the central question of this study?**
  Can combined therapy with mitochondrial transplantation, mitoquinone and melatonin effectively reduce myocardial ischaemia–reperfusion injury in aged rats?
- **What is the main finding and its importance?**
  The triple therapy significantly improved myocardial function, reduced creatine kinase‐MB levels and enhanced mitochondrial function and biogenesis by modulating the sirtuin 1, peroxisome proliferator‐activated receptor gamma coactivator 1‐alpha and nuclear respiratory factor 2 profiles, thereby providing substantial cardioprotection. This approach represents a promising therapeutic strategy for mitigating ischaemia–reperfusion injury, particularly in elderly patients who are at higher risk of cardiac damage.

## 1 INTRODUCTION

Ischaemic heart disease poses a growing global concern, particularly as cases increase among the elderly population (Heusch, 2020). The primary treatment for ischaemic heart disease involves reperfusion therapy aimed at restoring blood flow to the myocardial ischaemic zone. However, reperfusion can exacerbate myocardial cell death, known as lethal myocardial ischaemia–reperfusion (IR) injury (He et al., 2022). Ageing is a well‐known risk factor for ischaemic heart disease, affecting cellular signalling pathways that influence the development of IR injury and the effectiveness of cardioprotective treatments. This age‐related vulnerability to IR injury diminishes the efficacy of various ischaemic and pharmacological conditioning strategies in many experimental models and some clinical studies (Ferdinandy et al., 2023). Understanding the molecular mechanisms contributing to the susceptibility of the aged heart to ischaemic injury is crucial, because it informs the development of targeted therapies that mitigate IR injury and improve outcomes for elderly patients with ischaemic heart disease (Andreadou et al., 2018).

Animal studies have offered valuable insights into the mechanisms responsible for myocardial IR injury and have identified numerous potential therapeutic targets for cardioprotection. Nevertheless, translating these promising experimental findings into effective clinical treatments has posed significant challenges (Hausenloy & Heusch, 2019). An ongoing concern is the reliance on animal models that might not accurately replicate the pathophysiology of human cardiac diseases. For instance, the lack of inclusion of aged animals in experimental studies could restrict the applicability of the results to clinical populations, which typically consist of middle‐aged individuals with multiple co‐morbidities and who are taking multiple medications (Schulz et al., 2020). Moreover, numerous studies have focused exclusively on a single therapeutic approach aimed at a specific target within cardiomyocytes, which has demonstrated ineffectiveness in clinical practice (Davidson et al., 2019). Thus, when designing animal studies, it is crucial to model the disease as closely as possible to human conditions, adopt combination therapeutic strategies and focus on the most effective cellular mediators for manipulation. This approach ensures that findings from animal studies can be translated more effectively to human patients, potentially enhancing both the feasibility and the reliability of multitarget combination therapies in clinical practice (Davidson, Ferdinandy et al., 2019; Heusch, 2018).

Mitochondria play a critical role in cardioprotection, but ageing results in a decline in their normal number and function (a hypothesis of ageing). Consequently, in aged hearts, mitochondria are not in an ideal state (Guo et al., 2023; Miwa et al., 2022). Given that myocardial IR injury is multifaceted, single‐drug or single‐target interventions have been reported to address reperfusion injury inadequately (Davidson, Ferdinandy et al., 2019). Therefore, future studies should emphasize the exploration of multi‐target combination therapies to target the diverse components of this injury effectively (Li et al., 2022). Both mitoquinone (MitoQ; a potent mitochondria‐targeted antioxidant) and melatonin (acting as a cofactor in mitochondrial dynamics) have demonstrated positive effects on oxidative status, mitochondrial dynamics and biogenesis, in addition to autophagy/mitophagy activity in various disease conditions (Bermudez‐Gonzalez et al., 2022; Junior et al., 2018; Mao et al., 2020; Smith & Murphy, 2010; Sulaimon et al., 2022; Wang et al., 2024). Given the individual cardioprotective effects of MitoQ and melatonin, we hypothesize that combining these agents as a pre‐ and postconditioning strategy might exert superior cardioprotection. By leveraging their complementary mechanisms of action, we propose that a combined therapy might enhance cardioprotection beyond what can be achieved with individual treatments, potentially restoring ageing‐related loss of cardioprotection (Davidson, Ferdinandy et al., 2019; Krzywonos‐Zawadzka et al., 2017; Li et al., 2022). This combined approach aligns with the recommendations of the European Union (EU)‐CARDIOPROTECTION Cooperation in Science and Technology (COST) Action (CA16225), which underscores the necessity of investigating combined treatment strategies to enhance clinical outcomes (Hausenloy & Heusch, 2019; Lecour et al., 2021).

The ageing reperfused myocardium is characterized by dysfunctional mitochondria with accumulated mitochondrial DNA mutations. Consequently, merely administering mitoprotective agents might not be sufficient to address fully the complex mitochondrial deficits in aged cardiac tissue. A more comprehensive approach is needed, one that not only protects existing mitochondria but also improves both their quantity and function. In this context, mitochondrial transplantation (mitotherapy) emerges as a promising cardioprotective strategy. This innovative approach involves introducing healthy mitochondria into cardiomyocytes to replace or repair those damaged by ageing and IR injury (Bafadam et al., 2024; Dabravolski et al., 2022; Mokhtari & Badalzadeh, 2023; Quan et al., 2020). When combined with mitochondrial‐targeted antioxidants, such as MitoQ, and multifaceted agents, such as melatonin, which act as mitochondrial boosters, this integrative approach might offer enhanced prevention of myocardial IR injury in the context of ageing. However, further rigorous studies are necessary to validate this hypothesis and elucidate the underlying mechanisms. Such research would not only confirm the potential of this multifaceted approach but also provide crucial insights into optimizing cardioprotective strategies for aged hearts.

Given that ageing changes cellular conditions in ways that weaken myocardial function and worsen IR injury, and considering the limitations of single therapies in the context of ageing (Dong et al., 2020), we propose that a ‘mitochondrial cocktail’ consisting of these three key components (MitoQ, melatonin and mitochondrial transplantation) could be more effective than individual treatments and might represent a promising advancement in this field. Thus, the aim of this research was to examine how preconditioning with MitoQ, combined with postconditioning using melatonin and mitochondrial transplantation, impacts myocardial IR injury in aged rats, investigating whether changes in mitochondrial function and biogenesis could explain the beneficial effects of this triple combination approach.

## 2 MATERIALS AND METHODS

### 2.1 Ethical approval

All animal procedures adhered strictly to the guidelines outlined in the 8th Edition of the *Guide for the Care and Use of Laboratory Animals* by the United States National Institutes of Health (National Research Council, 2011). Ethical approval for the study protocol was granted by the Ethics Committee of Tabriz University of Medical Sciences in Tabriz, Iran (Ethics Approval Number: IR.TBZMED.AEC.1402.031).

### 2.2 Animals

Aged male Wistar rats (*n* = 54, aged 22–24 months, weighing 400–450 g) were used for experimental grouping, and young male Wistar rats (*n *= 10, aged 8 weeks, weighing 180–200 g) served as donors for isolated mitochondria. All rats were obtained from the Animal Center at Tabriz University of Medical Sciences (Tabriz, Iran) and were housed in pathogen‐free cages in a designated animal room. They were maintained on a 12 h–12 h light–dark cycle (lights on from 07:00 AM to 19:00 PM), with the room temperature set at 25°C ± 2°C and the humidity at 55%. The rats were given free access to standard chow and water and were allowed a 7‐day acclimation period before the experimental procedures began.

### 2.3 In vivo experimental set‐up

Aged rats were randomly assigned to nine experimental groups, each comprising six rats for cardiac function assessment, followed by biochemical and molecular analyses. The groups were categorized as follows:

1. Sham: Aged rats underwent a sham procedure.
2. IR: Aged rats experienced 30 min of regional ischaemia followed by 24 h of reperfusion.
3. IR+MQ: Aged rats received MitoQ (10 mg/kg/day for 14 days; GC30416, 98% purity, GLPBIO Technology, USA), dissolved in 1% DMSO via intraperitoneal injection prior to ischaemia (Oskuye et al., 2024).
4. IR+Mel: Aged rats subjected to the IR intervention were administered an intraperitoneal injection of melatonin (10 mg/kg; Sigma‐Aldrich, USA), dissolved in 1% ethanol and diluted in 1 mL of saline, immediately upon the initiation of reperfusion (Mokhtari et al., 2023).
5. IR+MT: Aged rats subjected to the IR intervention were given an intramyocardial injection of 500 µL of respiration buffer, which contained 6 × 10^6^ ± 5 × 10^5^ mitochondria/mL of respiration buffer at five sites within the peri‐infarct region immediately upon the initiation of reperfusion (Bafadam et al., 2024).
6. IR+MQ+Mel: Aged rats subjected to the IR intervention were given MitoQ and melatonin at their designated time points.
7. IR+MQ+MT: Aged rats subjected to the IR intervention were given MitoQ and mitochondrial transplantation at their designated time points.
8. IR+Mel+MT: Aged rats subjected to the IR intervention were given melatonin and mitochondrial transplantation at their designated time points.
9. IR+MQ+Mel+MT: Aged rats subjected to the IR intervention were given MitoQ, melatonin and mitochondrial transplantation at their designated time points.

**FIGURE 1:**
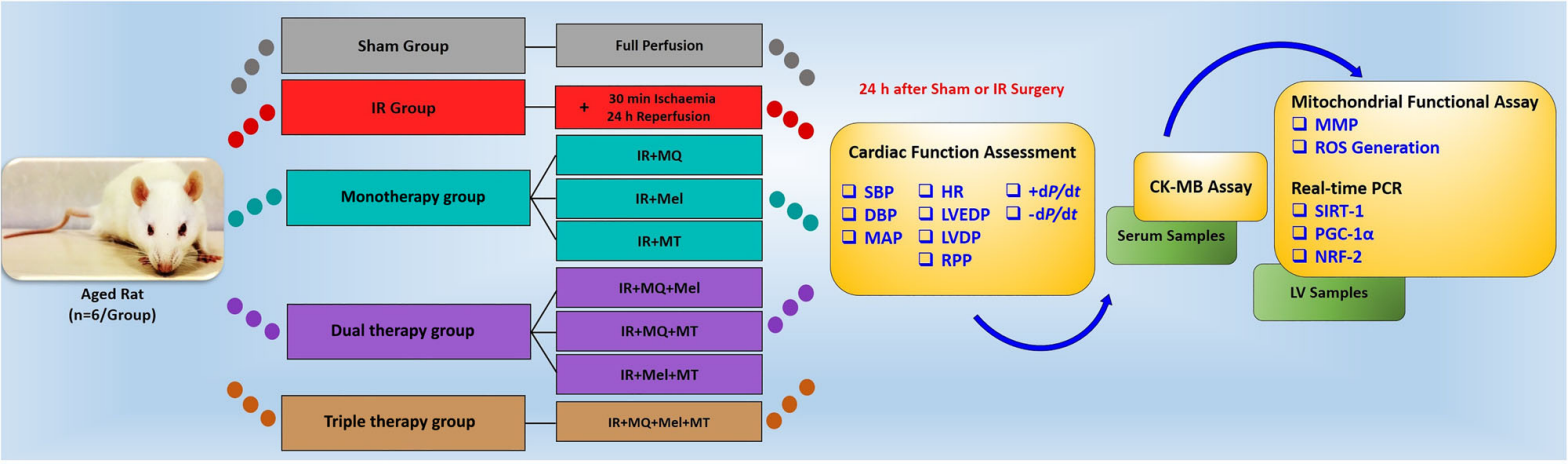
Schematic representation of the experimental design. In this study, aged rats were divided into nine groups, with or without IR, receiving MitoQ, melatonin, mitochondrial transplantation or various combinations of these treatments (six rats per group). The IR‐treated groups underwent 30 min of ischaemia followed by 24 h of reperfusion. The Sham group experienced open‐chest surgery without induction of myocardial IR injury and was monitored for the same duration as the ischaemia and reperfusion phases. MitoQ was administered intraperitoneally for 14 days prior to ischaemia, whereas melatonin was injected intraperitoneally upon the initiation of reperfusion. For mitochondrial transplantation, isolated mitochondria were injected intramyocardially at the start of the reperfusion phase. Cardiac function was assessed 24 h after the sham or IR procedures. Cardiac blood samples were then collected, and the hearts were removed under deep anaesthesia. Serum levels of CK‐MB were measured, and left ventricular tissues were harvested to evaluate mitochondrial function and gene expression. Abbreviations: *+*d*P*/d*t*, maximal rate of rise in left ventricular pressure; −d*P*/d*t*, maximal rate of decrease in left ventricular pressure; CK, creatine kinase; DBP, diastolic blood pressure; HR, heart rate; IR, ischaemia–reperfusion; LV, left ventricle; LVDP, left ventricular developed pressure; LVEDP, left ventricular end‐diastolic pressure; MAP, mean arterial pressure; Mel, melatonin; MMP, mitochondrial membrane potential; MQ, mitoquinone; MT, mitochondrial transplantation; *NRF‐2*, nuclear respiratory factor 2; *PGC‐1α*, peroxisome proliferator‐activated receptor gamma coactivator 1‐alpha; ROS, reactive oxygen species; RPP, rate–pressure product; SBP, systolic blood pressure; *SIRT‐1*, sirtuin 1.

Our previous studies clearly established the intervention doses, which were selected carefully based on the results of a pilot dose–response investigation and well‐documented literature demonstrating their significant impact on tissue protection (Bafadam et al., 2024; Mokhtari et al., 2023; Oskuye et al., 2024). The present study continues with the same proven doses in order to investigate their impact rigorously. It is important to note that both the Sham and IR groups were given equivalent amounts of DMSO, ethanol, saline and respiration buffer, without the isolated mitochondria, as their vehicle. Twenty‐four hours after the sham or IR procedure, rats were anaesthetized via intraperitoneal injection of ketamine (60 mg/kg; Sigma‐Aldrich, USA) and xylazine (10 mg/kg; Sigma‐Aldrich, USA) for assessment of cardiac function. After this evaluation, blood samples were collected, and the hearts were excised under deep general anaesthesia, which euthanized the rats, in accordance with the approved animal care protocols of Tabriz University of Medical Sciences. Blood samples were centrifuged to isolate serum, which was stored at −80°C for subsequent measurement of creatine kinase (CK)‐MB levels. Heart tissues were collected for further analysis: a segment of the area at risk from each heart was rapidly harvested for mitochondrial function assessment (*n* = 6/group), and another portion was frozen in liquid nitrogen and stored at −80°C for gene expression analysis (*n* = 5 per group) (Figure 1).

### 2.4 Mitochondria isolation and transplantation protocol

The methodology used to isolate mitochondria closely followed our prior research approaches (Bafadam et al., 2024). In summary, donor rats were anaesthetized with an intraperitoneal injection of ketamine (60 mg/kg) and xylazine (10 mg/kg). A biopsy punch was used to obtain a 40 mg sample of the pectoralis major muscle. After the muscle biopsy, the donor rats were euthanized under deep general anaesthesia by cervical dislocation, in accordance with the approved animal care protocols of Tabriz University of Medical Sciences.

The muscle biopsy was dissected and homogenized in an ice‐cold isolation buffer at 4°C. This buffer contained 2 mmol EDTA, 10 mmol HEPES, 70 mmol sucrose and 200 mmol mannitol, at a pH of 7.4. The tissue was homogenized using a precooled 2.0 mL Dounce homogenizer, with ∼1 mL of buffer per 15 mg of tissue. After homogenization, the mixture underwent centrifugation in two stages: initially at 1300*g* for 10 min to separate the supernatant from the homogenate, followed by a second step at 12,000*g* for 10 min to obtain the mitochondrial pellet. The mitochondrial pellet was resuspended in 100 µL of respiration solution containing 0.08 mmol ADP, 1 mmol ATP, 1 mmol dithiothreitol, 2 mmol dipotassium phosphate, 5 mmol sodium succinate, 10 mmol HEPES and 250 mmol sucrose (pH 7.4), then used for transplantation.

The protein concentration was determined using the bicinchoninic acid method, following the instructions specified by the manufacturer (Sigma‐Aldrich, USA) and using bovine serum albumin as a standard reference. Mitochondrial quantification was performed using haemocytometry (Preble et al., 2014).

Mitochondrial transplantation involved injecting 500 µL of mitochondrial suspension, containing 6 × 10^6 ^± 5 × 10^5^ mitochondria/mL of respiration buffer, at five distinct sites within the peri‐infarct region of the myocardium, corresponding to clock face positions at 10, 2, 7, 5 and 12 o'clock, to ensure optimal distribution. It should be noted that our previous research confirmed the successful internalization of labelled mitochondria into heart tissue following injection, as demonstrated by fluorescence microscopy. In the present work, we have built upon these findings by investigating mitochondrial transplantation combined with MitoQ and melatonin in myocardial IR injury in aged rats. We did not include the MitoTracker staining images in this study, because the results were published previously (Mokhtari & Badalzadeh, 2023).

### 2.5 Preparation of a myocardial IR injury model

Rats were anaesthetized with an intraperitoneal injection of a ketamine and xylazine mixture at doses of 60 mg/kg of ketamine and 10 mg/kg of xylazine. Following this, they were positioned on a heating pad to ensure that their body temperature remained ∼37°C. After endotracheal intubation, the rats were connected to a small animal ventilator (Harvard Apparatus VentElit, USA) for mechanical ventilation with room air. The settings were calibrated to provide a tidal volume of 2–3 mL/kg and a respiratory rate of 65–70 breaths/min. An incision was made in the left thorax at the fourth intercostal space, providing temporary access to the heart. Ligation was carried out ∼1–2 mm downstream from the origin of the left coronary artery using a 6–0 silk suture. This action blocked the left anterior descending coronary artery, temporarily stopping blood flow to the corresponding myocardial area. The controlled ischaemic period lasted for 30 min, resulting in ischaemia in the targeted region. Following this, the ligature was removed, allowing for reperfusion of the affected myocardium for 24 h. Rats in the Sham group underwent a similar procedure, but without the ligation step. During the operation, the PowerLab data acquisition system (ADInstrument, Australia) monitored ST segment elevation in ECG lead II, which served as an indicator of myocardial ischaemic injury. Additionally, a noticeable regional paleness in the anterior wall of the left ventricle, immediately below the ligation site, was observed, indicating reduced blood flow and oxygenation to the affected myocardium. The surgical procedure was completed by closing the chest with a 2–0 silk suture. Once the animals regained independent respiration, they were extubated and allowed to recover. Haemodynamic parameters were then measured after this recovery period, as described in the following section. Postoperatively, rats were administered buprenorphine (0.05 mg/kg, subcutaneously) for pain management and tetracycline as an antibiotic.

### 2.6 Measurement of haemodynamic parameters

Twenty‐four hours after either the sham surgery or the IR intervention, rats were anaesthetized with an intraperitoneal injection of a ketamine and xylazine mixture, using doses of 60 mg/kg of ketamine and 10 mg/kg of xylazine. A PE50 catheter was inserted into the right carotid artery to measure haemodynamic parameters. This catheter was connected to a PowerLab data acquisition system (ADInstruments, Australia) via a pressure transducer, and data were analysed using LabChart v.7.3 software (ADInstruments, Australia). Systolic blood pressure (SBP, in millimetres of mercury), diastolic blood pressure (DBP, in millimetres of mercury) and mean arterial pressure (MAP, in millimetres of mercury) were recorded continuously over a period of 20 min. Heart rate (HR, in beats per minute) was also monitored concurrently. After an interval of 20 min, the catheter was gently inserted into the left ventricle to evaluate left ventricular functional parameters. These measurements included left ventricular end‐diastolic pressure (LVEDP, in millimetres of mercury), left ventricular developed pressure (LVDP, in millimetres of mercury), rate–pressure product (RPP, in millimetres of mercury multiplied by beats per minute), maximal rate of rise in left ventricular pressure (*+*d*P*/d*t*, in millimetres of mercury per second) and maximal rate of decrease in left ventricular pressure (−d*P*/d*t*, in millimetres of mercury per second). The LVDP was computed as the difference between LVEDP and left ventricular systolic pressure (LVSP, in millimetres of mercury). The RPP, serving as a measure of cardiac contractility, was determined using the following formula: RPP = LVDP × HR. After the haemodynamic assessments, blood samples were taken from the heart, and the heart was removed under deep general anaesthesia, which euthanized the rats, in accordance with the approved animal care protocols of Tabriz University of Medical Sciences. Heart and serum samples were subsequently processed for further analysis.

### 2.7 Serum biochemical analysis

Cardiac blood samples were collected at the end of the haemodynamic assessments as described above and placed in non‐anticoagulant tubes at 25°C for 2 h. Following this, the samples underwent centrifugation at 3000*g* for 10 min to separate the serum. CK‐MB levels in the serum were assessed using an enzyme‐linked immunosorbent assay (ELISA) with a commercial kit (Pars Azmoon Company, Tehran, Iran), following the guidelines provided. The findings are reported in international units per litre.

### 2.8 Evaluation of mitochondrial functional parameters

As previously described, we used differential centrifugation to isolate mitochondria from freshly obtained myocardial tissue samples from the area at risk in order to evaluate cardiac mitochondrial functional parameters, such as mitochondrial membrane potential and reactive oxygen species (ROS) generation (Bayrami et al., 2018).

The approach for evaluating mitochondrial membrane potential in isolated mitochondria included using the fluorescent dye JC‐1 (5,5′,6,6′‐tetrachloro‐1,1′,3,3′‐tetraethylbenzimidazolylcarbocyanine iodide). The evaluation followed precise instructions from the mitochondrial membrane potential assay kit (Sigma‐Aldrich, USA). In summary, 5 µg of isolated mitochondria were gently suspended in a 100 µL assay buffer containing 0.2 µg/mL JC‐1. The mixture was then incubated in darkness at room temperature for 10–20 min. JC‐1 aggregates, which emit red fluorescence in healthy cells, were excited at a wavelength of 525 nm and detected at an emission wavelength of 590 nm. In contrast, JC‐1 monomers, which emit green fluorescence in cells under stress, were excited at 490 nm and detected at an emission wavelength of 530 nm. Fluorescence measurements were conducted with a spectrofluorometer (FP‐750, Japan), and changes in mitochondrial potential were analysed using the ratio of red to green fluorescence intensities. Decreases in this ratio indicate mitochondrial membrane depolarization. Data are presented as fluorescence units per microgram of protein.

To investigate mitochondrial ROS generation, the mitochondrial pellets were exposed to a 2 µM solution of 2′,7′‐dichlorofluorescein diacetate (DCFDA) dye for 30 min at room temperature. After DCFDA diffuses into the organelle, ROS induce its oxidation, resulting in the formation of a highly fluorescent compound known as 2′,7′‐dichlorofluorescein (DCF). Using a fluorometric approach, the fluorescence from DCF was detected with excitation at 480 nm and emission at 530 nm. Increased fluorescent intensity per milligram of protein in the samples was correlated directly with heightened mitochondrial ROS generation levels.

### 2.9 Assessment of gene expression

Real‐time PCR was used to evaluate the expression of sirtuin 1 (*SIRT‐1*), peroxisome proliferator‐activated receptor gamma coactivator 1‐alpha (*PGC‐1α*) and nuclear respiratory factor 2 (*NRF‐2*) genes. Total RNA was extracted from myocardial tissue samples of the area at risk using TRIzol reagent (Invitrogen, Carlsbad, CA, USA). The extracted RNA was then converted into complementary DNA using the PrimeScript RT reagent kit (Takara, Japan), following the manufacturer's guidelines. Quantitative analysis of the target mRNA expression levels was conducted using real‐time PCR with a SYBR Green‐based detection system. The relative expression levels of the target mRNAs were quantified using the Livak comparative *CT* method, and these values were then normalized by referencing the transcript levels of the housekeeping gene glyceraldehyde 3‐phosphate dehydrogenase (*GAPDH*). The primer sequences used for the real‐time PCR analyses are provided in Table 1.

**TABLE 1:** The sequences of primers used in real‐time PCR.

Gene	Primer sequence
*SIRT‐1*	Forward: 5′‐GCACTAATTCCAAGTTCTATACCCCAT‐3′ Reverse: 5′‐CTCGCCACCTAACCTATGACACAA‐3′
*PGC‐1α*	Forward: 5′‐CTAGCGGTCCTCACAGAGACA‐3′ Reverse: 5′‐GTCAGGCATGGAGGAAGGAC‐3′
*NRF‐2*	Forward: 5′‐GCTGTGTGTTCTGAGTATCGT‐3′ Reverse: 5′‐TCATAATCCTTCTGTCGCTGA‐3′
*GAPDH*	Forward: 5′‐CAAGATCATCAGCAATGCCTCC‐3′ Reverse: 5′‐GCCATCACGCCAGTTTCC‐3′

Abbreviations: *GAPDH*, glyceraldehyde 3‐phosphate dehydrogenase; *NRF‐2*, nuclear respiratory factor 2; *PGC‐1α*, proliferator‐activated receptor gamma coactivator 1‐alpha; *SIRT‐1*, sirtuin 1.

### 2.10 Statistical analysis

The experimental findings underwent statistical analysis using a one‐way ANOVA, followed by Tukey's *post hoc* test, executed with GraphPad Prism (GraphPad Software, v.9.5.1, San Diego, CA, USA). The data were expressed as the mean ± SD. A value of *P < *0.05 was considered statistically significant.

## 3 RESULTS

### 3.1 Triple therapy enhanced cardiac function in aged rats with myocardial IR injury

Evaluating the effects of triple combination therapy on cardiovascular parameters in aged rats with myocardial IR injury is essential for determining its clinical relevance and therapeutic efficacy.

#### 3.1.1 Arterial blood pressure profiles

**FIGURE 2:**
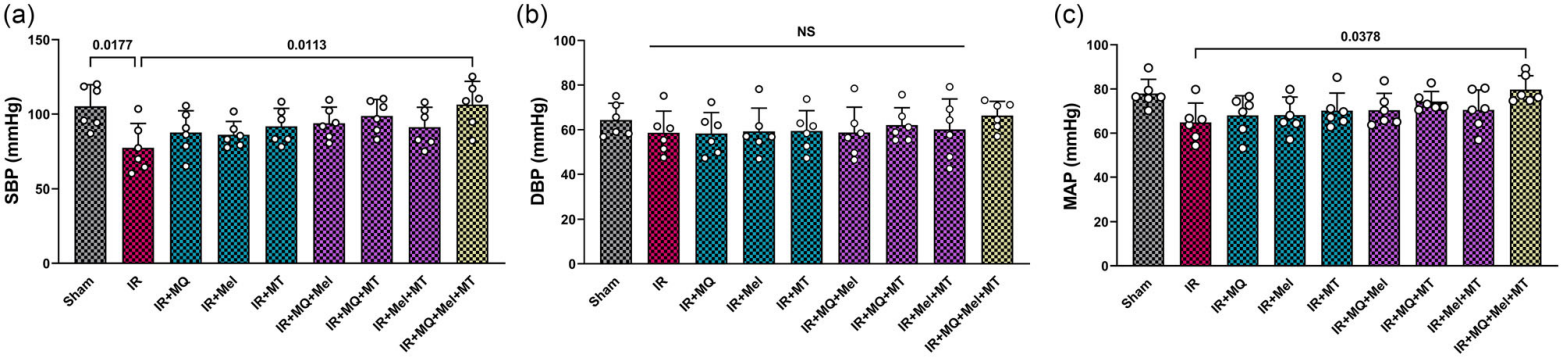
The impact of mitoquinone, melatonin and mitochondrial transplantation, individually and in combinations (dual and triple), on SBP (a), DBP (b) and MAP (c) across different groups (six rats per group). The data are presented as the mean ± SD. Abbreviations: DBP, diastolic blood pressure; IR, ischaemia–reperfusion; MAP, mean arterial pressure; Mel, melatonin; MQ, mitoquinone; MT, mitochondrial transplantation; NS, not significant; SBP, systolic blood pressure.

As depicted in Figure 2, the experimental groups exhibited distinct patterns in their arterial blood pressure metrics. In comparison to the Sham group, the induction of myocardial IR injury in aged rats led to a significant decrease in SBP (*P *= 0.0117; Figure 2a). When applied individually or in pairs, MitoQ, melatonin and mitochondrial transplantation have no significant effects on SBP, DBP or MAP in comparison to untreated aged rats (Figure 2). However, the application of MitoQ, melatonin and mitochondrial transplantation in a triple combination resulted in a significant elevation in SBP and MAP in comparison to the untreated aged rats (*P* = 0.0113 and *P *= 0.0378, respectively; Figure 2a,c).

#### 3.1.2 Cardiac haemodynamic profiles

**FIGURE 3:**
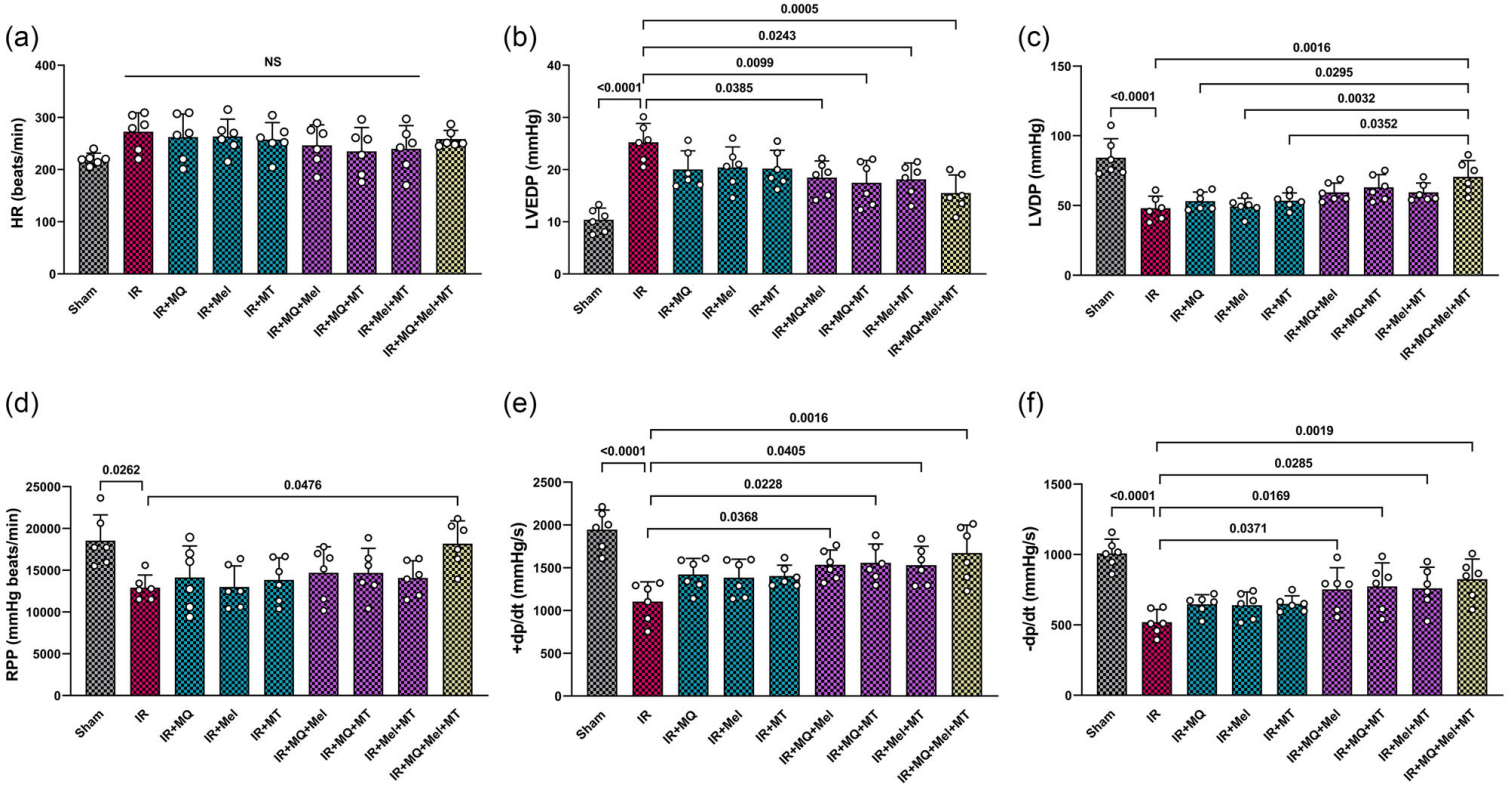
The impact of mitoquinone, melatonin and mitochondrial transplantation, individually and in combinations (dual and triple), on HR (a), LVEDP (b), LVDP (c), RPP (d), *+*d*P*/d*t* (e) and −d*P*/d*t* (f) across different groups (six rats per group). The data are presented as the mean ± SD. Abbreviations: *+*d*P*/d*t*, maximal rate of rise in left ventricular pressure; −d*P*/d*t*, maximal rate of decrease in left ventricular pressure; HR, heart rate; IR, ischaemia–reperfusion; LVDP, left ventricular developed pressure; LVEDP, left ventricular end‐diastolic pressure; Mel, melatonin; MQ, mitoquinone; MT, mitochondrial transplantation; RPP, rate–pressure product.

The data presented in Figure 3a–d illustrate the changes in HR and key left ventricular functional parameters, including LVEDP, LVDP and RPP across the experimental groups. As shown in Figure 3a, the heart rates did not exhibit statistically significant differences across the experimental groups. In Figure 3b–d, the experimental group subjected to myocardial IR injury exhibited notable alterations in left ventricular functional parameters in comparison to the Sham group. Specifically, LVEDP showed a significant increase (*P* < 0.0001), whereas LVDP and RPP showed significant decreases (*P < *0.0001 and *P* = 0.0262, respectively). The individual therapeutic interventions failed to elicit significant improvements in LVEDP, LVDP and RPP in comparison to the IR group (Figure 3b–d). The use of MitoQ, melatonin or mitochondrial transplantation in dual combinations led to a notable reduction in LVEDP in comparison to the IR group (*P = *0.0385, *P = *0.0099 and *P = *0.0243, respectively; Figure 3b). Triple combination of therapeutic interventions led to a statistically significant reduction in LVEDP (*P = *0.0005), alongside significant elevations in LVDP (*P = *0.0016) and RPP (*P = *0.0476), in comparison to the untreated IR group (Figure 3b–d). Furthermore, the combined conditioning with MitoQ, melatonin and mitochondrial transplantation in a triple manner led to a notable enhancement in the LVDP compared with using MitoQ alone (*P = *0.0295), melatonin alone (*P = *0.0032) or mitochondrial transplantation alone (*P = *0.0352; Figure 3c).

Figure 3e,f depicts the differences in *+*d*P*/d*t* and −d*P*/d*t* (across all experimental groups). In the group that underwent myocardial IR injury, there was a notable decrease in both *+*d*P*/d*t* and −d*P*/d*t* in comparison to the Sham group, with statistical significance observed (*P < *0.0001 for both). The individual therapeutic interventions had no significant effects on *+*d*P*/d*t* and −d*P*/d*t* when compared with the IR group. In contrast, the groups that underwent dual treatments demonstrated marked haemodynamic alterations relative to the IR group. Specifically, the combinations of MitoQ with melatonin, MitoQ with mitochondrial transplantation, and melatonin with mitochondrial transplantation resulted in significant increases in both +d*P*/d*t* (*P =* 0.0368, *P =* 0.0228, and *P =* 0.0405, respectively) and −d*P*/d*t* (*P =* 0.0371, *P =* 0.0169, and *P =* 0.0285, respectively). Remarkably, the triple treatment demonstrated a more pronounced increase in both *+*d*P*/d*t* and −d*P*/d*t* compared with the untreated IR group, with statistical significance being observed (*P = *0.0016 and *P = *0.0019, respectively; Figure 3e,f).

### 3.2 Triple therapy decreased CK‐MB serum levels in aged rats with myocardial IR injury

**FIGURE 4:**
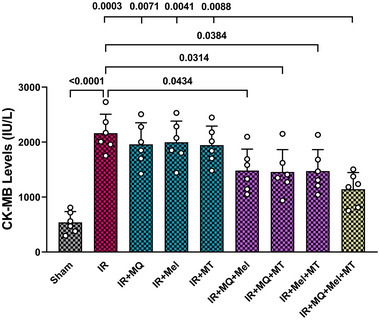
The impact of mitoquinone, melatonin and mitochondrial transplantation, individually and in combinations (dual and triple), on CK‐MB serum levels across different groups (six rats per group). The data are presented as the mean ± SD. Abbreviations: CK, creatine kinase; IR, ischaemia–reperfusion; Mel, melatonin; MQ, mitoquinone; MT, mitochondrial transplantation.

CK‐MB serves as a cardiac biomarker indicating myocardial damage. In the present study, CK‐MB serum levels were measured and compared among the experimental groups to evaluate the efficacy of triple combination therapy in reducing myocardial damage following IR injury in aged rats. Figure 4 shows the changes in CK‐MB serum levels among all experimental groups. Myocardial IR injury in aged rats led to a significant rise in serum CK‐MB levels in comparison to the Sham group (*P < *0.0001). The single therapies did not significantly affect serum CK‐MB levels in comparison to the Sham group. The use of dual treatments, specifically MitoQ plus melatonin, MitoQ plus mitochondrial transplantation, and melatonin plus mitochondrial transplantation, led to a notable reduction in serum CK‐MB levels when compared with the untreated IR group (*P = *0.0434, *P = *0.0314 and *P  = *0.0384, respectively). Furthermore, the triple combination treatment was significantly more effective in reducing CK‐MB serum levels compared to the untreated IR group (*P = *0.0003) and the groups that received monotherapies with MitoQ, melatonin, or mitochondrial transplantation (*P = *0.0071, *P = *0.0041 and *P = *0.0088, respectively; Figure 4).

### 3.3 Triple therapy improved mitochondrial function in aged IR hearts

**FIGURE 5:**
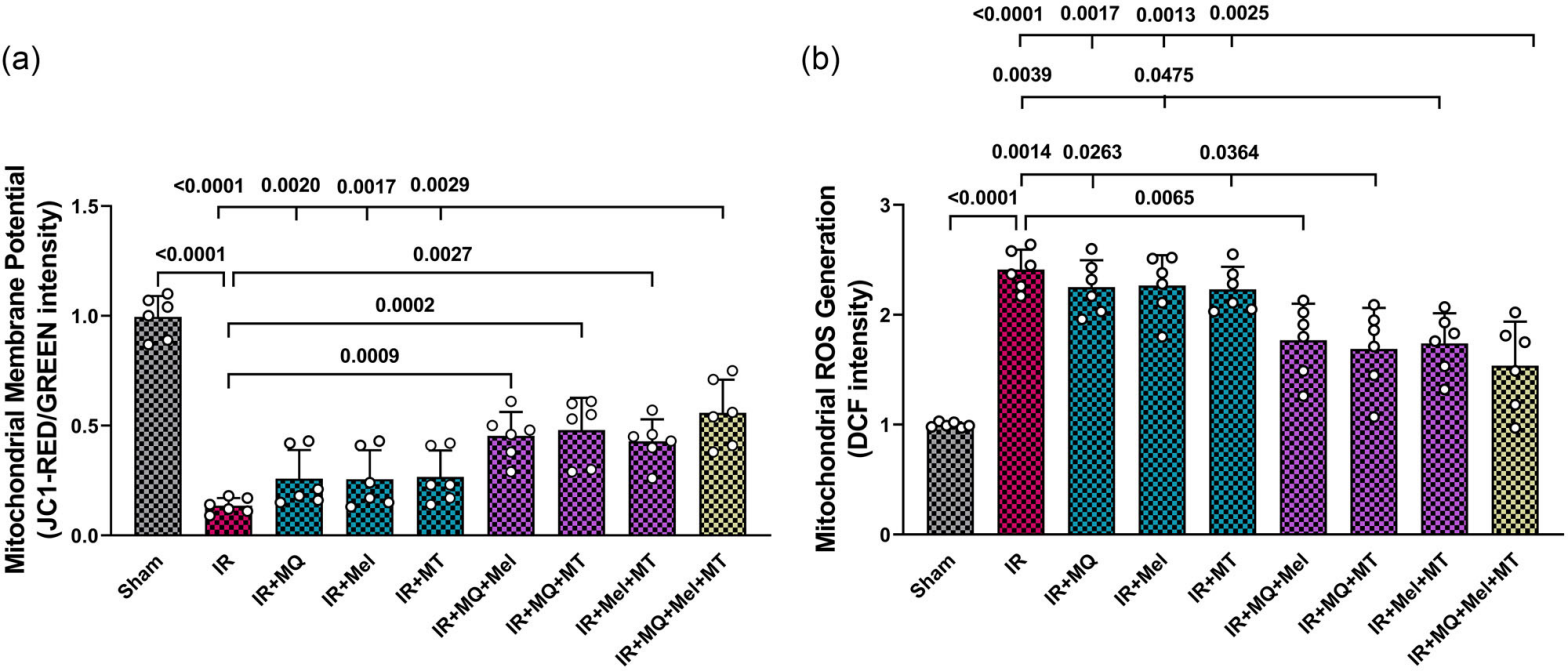
The impact of mitoquinone, melatonin and mitochondrial transplantation, individually and in combinations (dual and triple), on mitochondrial membrane potential alterations (a) and mitochondrial ROS generation (b) across different groups (six rats per group). The data are presented as the mean ± SD. Abbreviations: DCF, 2′,7′‐dichlorofluorescein; IR, ischaemia–reperfusion; JC‐1, 5,5′,6,6′‐tetrachloro‐1,1′,3,3′‐tetraethylbenzimidazolylcarbocyanine iodide; Mel, melatonin; MQ, mitoquinone; MT, mitochondrial transplantation; ROS, reactive oxygen species.

In the present study, two aspects of mitochondrial function that were evaluated were mitochondrial membrane potential (measured by JC‐1 red/green intensity) and ROS generation (measured by DCF intensity). Figure 5 illustrates that aged rats subjected to myocardial IR injury demonstrated a significant reduction in the red/green ratio, indicating compromised or diminished membrane potential, along with elevated mitochondrial ROS generation in comparison to the Sham group (*P < *0.0001 for both). Although monotherapies did not significantly impact these mitochondrial functional endpoints, their combined dual application, specifically MitoQ plus melatonin, MitoQ plus mitochondrial transplantation, and melatonin plus mitochondrial transplantation, significantly enhanced mitochondrial membrane potential (*P = *0.0009, *P = *0.0002 and *P = *0.0027, respectively) and decreased mitochondrial ROS generation (*P = *0.0065, *P = *0.0014 and *P = *0.0039, respectively) compared with the IR group (Figure 5a,b). Furthermore, the combined use of MitoQ and mitochondrial transplantation significantly reduced mitochondrial ROS generation in comparison to the groups that received them individually (*P = *0.0263 vs. IR+MQ group and *P = *0.0364 vs. IR+MT group; Figure 5b). Also, combined use of melatonin and mitochondrial transplantation led to a significant decrease in mitochondrial ROS generation in comparison to the group that received melatonin alone (*P = *0.0475; Figure 5b). The triple combination treatment significantly improved mitochondrial membrane potential in comparison to both the untreated IR group (*P < *0.0001) and the groups that received single therapies with MitoQ, melatonin or mitochondrial transplantation (*P = *0.0020, *P = *0.0017 and *P = *0.0029, respectively; Figure 5a). Additionally, it significantly reduced mitochondrial ROS generation in comparison to the untreated IR group (*P < *0.0001) and the groups that received monotherapies with MitoQ, melatonin or mitochondrial transplantation (*P = *0.0017, *P = *0.0013 and *P = *0.0025, respectively; Figure 5b).

### 3.4 Triple therapy enhanced mitochondrial biogenesis gene expression in aged IR hearts

**FIGURE 6:**
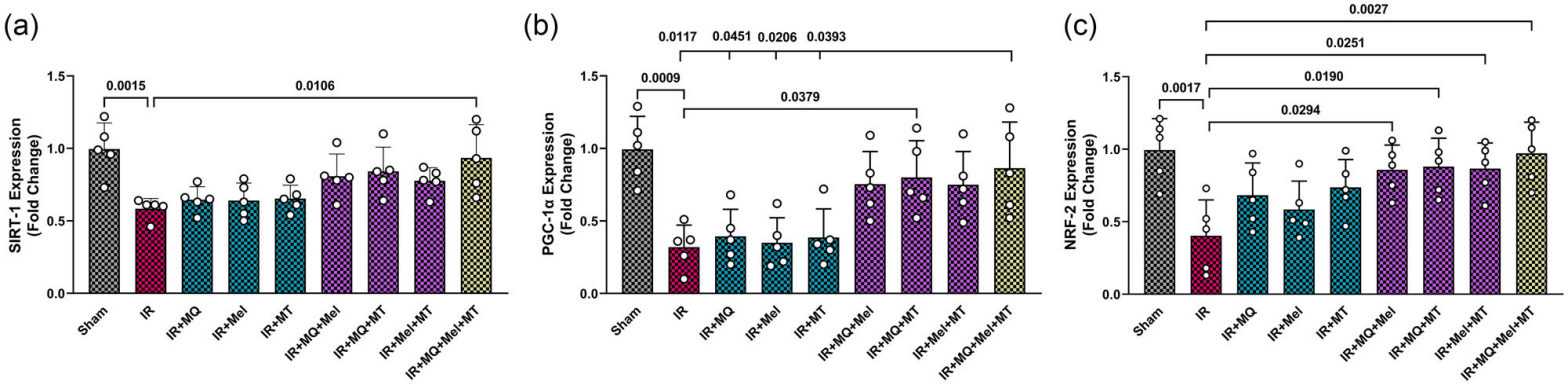
The impact of mitoquinone, melatonin and mitochondrial transplantation, both individually and in combinations (dual and triple), on myocardial mRNA levels of *SIRT‐1* (a), *PGC‐1α* (b) and *NRF‐2* (c) across different groups (five rats per group). The data are presented as the mean ± SD. Abbreviations: IR, ischaemia–reperfusion; Mel, melatonin; MQ, mitoquinone; MT, mitochondrial transplantation; *NRF‐2*, nuclear respiratory factor 2; *PGC‐1α*, peroxisome proliferator‐activated receptor gamma coactivator 1‐alpha; *SIRT‐1*, sirtuin 1.

Given that we investigated the effects of triple therapy on cardiac mitochondrial function following IR injury, assessing the expression of key genes involved in mitochondrial biogenesis (such as *SIRT‐1*, *PGC‐1α* and *NRF‐2*) provided essential insights into the underlying mechanisms by which this triple therapy might exert its mitoprotective effects. As shown in Figure 6, it is evident that the mRNA expression levels of *SIRT‐1*, *PGC‐1α* and *NRF‐2* were significantly reduced in the IR group compared with the Sham group (*P = *0.0015, *P = *0.0009 and *P = *0.0017, respectively). Individual applications of MitoQ, melatonin or mitochondrial transplantation did not significantly affect the mRNA expression levels of *SIRT‐1*, *PGC‐1α* and *NRF‐2* compared with the untreated IR group (Figure 6a‐c). However, dual combinations, specifically MitoQ plus melatonin, MitoQ plus mitochondrial transplantation, and melatonin plus mitochondrial transplantation, led to a significant upregulation of *NRF‐2* mRNA expression levels compared with the IR group (*P = *0.0294, *P = *0.0190 and *P = *0.0251, respectively; Figure 6c). Furthermore, the combined application of MitoQ and mitochondrial transplantation significantly increased the mRNA expression levels of *PGC‐1α* in comparison to the untreated IR group (*P = *0.0379; Figure 6b). Notably, the triple combination therapy resulted in a statistically significant increase in the mRNA expression of *SIRT‐1*, *PGC‐1α* and *NRF‐2* genes when compared with the IR group (*P = *0.0106, *P = *0.0117 and *P = *0.0027, respectively; Figure 6a‐c). Furthermore, this triple combination treatment demonstrated a more robust positive effect on the *PGC‐1α* mRNA expression levels in comparison to the groups receiving monotherapies with MitoQ, melatonin or mitochondrial transplantation (*P = *0.0451, *P = *0.0206 and *P = *0.0393, respectively; Figure 6b).

## 4 DISCUSSION

The study results highlight the promising therapeutic potential of a novel mitochondria‐targeted combination therapy that includes MitoQ, melatonin and mitochondrial transplantation in mitigating myocardial IR injury in aged rats. This comprehensive treatment strategy involved preconditioning with MitoQ, followed by postconditioning with melatonin and mitochondrial transplantation, resulting collectively in notable enhancements in myocardial function and reductions in serum CK‐MB levels post‐IR injury. Although individual administration of MitoQ, melatonin or mitochondrial transplantation did not exhibit significant cardioprotective effects against myocardial IR injury, their combined use in dual combinations showed modest beneficial effects on certain parameters. Interestingly, some of the dual treatments demonstrated stronger effects compared with individual treatments, suggesting potential additive interactions between them in mitigating cardiac damage in the context of IR injury. However, the most remarkable outcomes were observed with the triple combination therapy, in which the combined application of MitoQ, melatonin and mitochondrial transplantation exerted substantial cardioprotective effects against IR injury in aged hearts. The effectiveness of the triple therapy surpassed that of both individual and dual therapies, highlighting an additive and potent effect of the combined mitochondrial cocktail in preserving myocardial function and reducing injury in the setting of IR. The cardioprotective effects of the mitochondria‐targeted combination therapy seem to be linked to improvements in mitochondrial function and the upregulation of the *SIRT‐1*/*PGC‐1α*/*NRF‐2* profiles in aged myocardium. These molecular alterations promote mitochondrial biogenesis and overall mitochondrial function, thereby contributing to the maintenance of cardiac function and integrity in the context of IR injury.

Prior experimental studies have showcased the cardioprotective properties of MitoQ, melatonin and mitochondrial transplantation in young animal models, demonstrating their ability to alleviate the adverse effects of IR injury (Guariento et al., 2020; Junior et al., 2018; Masuzawa et al., 2013; Wang et al., 2024). In our present investigation, we opted for an aged animal model to better reflect the real‐world scenario, given that most individuals encountering myocardial IR injury are elderly. Intriguingly, when administered individually, MitoQ, melatonin and mitochondrial transplantation did not confer cardioprotective benefits in the aged animal model, in contrast to the results observed in younger animals. This discrepancy might be attributed to the fact that earlier research predominantly used young and healthy animal models devoid of risk factors or co‐morbidities, whereas our study involved aged rats (Lecour et al., 2021). Ageing is linked to various unfavourable biological alterations in cardiomyocytes, including heightened oxidative stress, mitochondrial dysfunction and disrupted cellular signalling pathways, all of which could render the heart more vulnerable to IR injuries, thereby diminishing the efficacy of the tested interventions against myocardial IR injury (Ferdinandy et al., 2023). In our study, the individual application of MitoQ, melatonin and mitochondrial transplantation failed to enhance heart function or decrease CK‐MB levels significantly. This might be attributed to the cumulative effects of ageing, which can critically alter the activity of molecular salvage signalling pathways and mediators essential for cytoprotection (Randhawa et al., 2018). The dual use of these interventions showed positive effects on certain but not all measured endpoints. However, the most compelling discovery was that the triple combination of MitoQ, melatonin and mitochondrial transplantation managed to overcome the limitations observed with individual and dual applications. This indicates that the multi‐target approach effectively addressed the intricate and interconnected pathways affected by ageing in the heart. By simultaneously targeting multiple mechanisms, the triple combination delivered a more comprehensive and potent cardioprotective effect. These results underscore the significance of considering multi‐target strategies, particularly in the context of age‐related cardiac dysfunction, where underlying pathways might be highly intricate and intertwined (Mokhtari & Badalzadeh, 2023; Mokhtari et al., 2023). The synergistic or additive effects of the triple combination suggest that this approach could be a more promising strategy for enhancing heart function and mitigating the adverse effects of ageing on the cardiovascular system.

To investigate the mechanisms underlying the enhanced cardioprotection conferred by the triple combination therapy in aged rats following myocardial IR injury, we evaluated its impact on mitochondrial function and biogenesis. Mitochondrial dysfunction plays a pivotal role in myocardial IR injury, driving processes such as impaired oxidative phosphorylation, excessive ROS generation and the opening of the mitochondrial permeability transition pore, which culminate in mitochondrial swelling, membrane depolarization and the release of pro‐apoptotic factors (Heusch, 2024; Sagris et al., 2024). Ageing intensifies these effects owing to cumulative mitochondrial DNA mutations, impaired mitochondrial biogenesis and dysregulated dynamics, rendering aged hearts particularly vulnerable to IR injury (Haas, 2019; Ruiz‐Meana et al., 2020). Our findings suggest that the combination therapy of MitoQ, melatonin and mitochondrial transplantation acts through complementary mechanisms. MitoQ, a mitochondria‐targeted antioxidant, is likely to exert its effects by neutralizing mitochondrial ROS and maintaining the integrity of the electron transport chain, which is critical for reducing oxidative damage and preserving mitochondrial membrane potential (Sulaimon et al., 2022). By administering MitoQ prior to ischaemia, we hypothesize that it creates a preconditioned myocardial environment with enhanced mitochondrial resilience, optimizing the response to subsequent interventions. Melatonin, delivered during reperfusion, is likely to contribute by modulating acute oxidative stress and inflammation. Its ability to upregulate antioxidant enzymes and inhibit pro‐inflammatory cytokine production might mitigate reperfusion‐associated injury. Additionally, the influence of melatonin on sirtuins, particularly *SIRT‐1*, might enhance mitochondrial biogenesis through pathways involving *PGC‐1α* and *NRF‐2*, as observed in our study (Bermudez‐Gonzalez et al., 2022; Zhai et al., 2017). Mitochondrial transplantation provides a unique and direct mechanism to restore bioenergetic capacity by replacing damaged mitochondria with healthy, functional ones. This approach addresses the long‐term consequences of ageing‐related mitochondrial deficits, including reduced ATP production and compromised mitochondrial quality control (Baran et al., 2024; Park et al., 2021). The synergistic effects observed in our study are likely to stem from the integration of these mechanisms, whereby MitoQ preconditions the myocardial environment, melatonin mitigates acute injury, and mitochondrial transplantation facilitates structural and functional recovery. Together, they target key aspects of mitochondrial dysfunction, providing a comprehensive strategy to enhance cardioprotection.

Although our study establishes a strong foundation, the exact molecular mechanisms driving the observed benefits warrant further investigation. For instance, exploration of the interplay between the transplanted mitochondria and host cell mitochondrial dynamics could provide additional insights. Moreover, elucidation of the signalling cascades activated by this combination therapy, such as the role of mitophagy or mitochondrial fusion/fission, could uncover new therapeutic targets. In summary, the combination therapy leverages multiple, complementary mechanisms to address the complexities of myocardial IR injury in aged hearts. Adhering to guidelines established by the EU‐CARDIOPROTECTION COST Action Group and conducting additional research can enhance the likelihood of translating our findings into tangible clinical benefits for patients (Lecour et al., 2021).

### 4.1 Limitations and suggestions

Our study demonstrates promising acute cardioprotective effects of the mitochondria‐targeting triple combination; however, several limitations warrant attention in future research. Although our 24 h endpoint aligns with the IMproving Preclinical Assessment of Cardioprotective Therapies (IMPACT) criteria for assessing acute effects, longer‐term follow‐up is essential to monitor potential development of cardiomyopathy and evaluate sustained benefits. Future studies should extend the observation period to ≥28 days to assess scar size relative to left ventricular mass and remodelling (Lecour et al., 2021). The use of a single species limits generalizability, necessitating research with diverse animal models to validate broader applicability. Further investigations are needed to elucidate specific molecular mechanisms, including mitochondrial dynamics, signalling pathways and protein expression profiles. Although the mitochondrial analyses add significant depth, incorporating additional histological techniques, such as immunohistochemistry to identify introduced mitochondria or electron microscopy to evaluate mitochondrial morphology, would enhance the understanding of the therapeutic effects observed. Dose–response studies for each component of the cocktail will help to optimize concentrations for maximum efficacy and minimal adverse effects. Expanding research to include models with concurrent conditions, such as diabetes or hypertension, will offer a more comprehensive understanding of the effectiveness and limitations of the therapy. Exploring different administration sequences could reveal more efficient treatment protocols. Thorough safety evaluations are essential to ensure safe clinical implementation. In line with IMPACT criteria, the treatment should undergo multicentre validation using diverse species and strains, including both male and female subjects (Lecour et al., 2021). Addressing these limitations and expanding our research scope will refine the therapeutic strategy and enhance its potential for successful clinical translation.

## 5 CONCLUSION

In summary, this study illustrated that pretreatment with MitoQ substantially boosted the cardioprotective effects of postconditioning with melatonin and mitochondrial transplantation in aged rats with myocardial IR injury. The combination of MitoQ preconditioning followed by melatonin and mitochondrial transplantation postconditioning not only reinstated complete cardioprotection in the aged rat myocardium but also outperformed the efficacy of individual or dual therapies. This enhanced cardioprotection was linked, in part, to the enhancement in mitochondrial function and biogenesis, facilitated by the upregulation of the *SIRT‐1*/*PGC‐1α*/*NRF‐2* profiles. Discovering additional shared protective mechanisms could provide further insights. These results underscore the potential therapeutic value of this mitochondrial cocktail and set the stage for future investigations into its mechanisms and possible clinical uses.

## AUTHOR CONTRIBUTIONS

Reza Badalzadeh and Samad Ghaffari were responsible for the study design and oversight of the entire project. Behnaz Mokhtari carried out the experimental procedures, gathered and analysed the data and wrote the manuscript. Mitra Delkhah assisted with the experimental procedures and data analysis. Reza Badalzadeh, Samad Ghaffari and Behnaz Mokhtari critically reviewed and completed the manuscript. All authors approved the final version of the manuscript and agree to be accountable for all aspects of the work in ensuring that questions related to the accuracy or integrity of any part of the work are appropriately investigated and resolved. All persons designated as authors qualify for authorship, and all those who qualify for authorship are listed. The authors confirm that all data were produced internally and that no paper mill was used.

## CONFLICT OF INTEREST

The authors have no conflicts of interest to disclose regarding the research, authorship and/or publication of this article.

## Data Availability

The datasets used during the present work are available from the corresponding authors on reasonable request.
